# Assessment of clinical trial protocols for pathology content using the SPIRIT‐Path guidelines highlights areas for improvement

**DOI:** 10.1002/cjp2.274

**Published:** 2022-05-31

**Authors:** Peter Robinson, Chris M Bacon, Shujing J Lim, Abeer M Shaaban, Daniel Brierley, Ian Lewis, David J Harrison, Timothy J Kendall, Max Robinson

**Affiliations:** ^1^ School of Medical Education Newcastle University Newcastle upon Tyne UK; ^2^ Translational and Clinical Research Institute Newcastle University Newcastle upon Tyne UK; ^3^ Department of Cellular Pathology Newcastle upon Tyne Hospitals NHS Foundation Trust Newcastle upon Tyne UK; ^4^ Department of Histopathology Queen Elizabeth Hospital Birmingham UK; ^5^ Institute of Cancer and Genomic Sciences University of Birmingham Birmingham UK; ^6^ Unit of Oral and Maxillofacial Pathology University of Sheffield Sheffield UK; ^7^ National Cancer Research Institute London UK; ^8^ School of Medicine University of St Andrews St Andrews UK; ^9^ Centre for Inflammation Research University of Edinburgh Edinburgh UK

**Keywords:** cellular pathology, clinical trials, protocols, guidance, checklist

## Abstract

The SPIRIT (Standard Protocol Items: Recommendations for Interventional Trials) 2013 Statement provides evidence‐based recommendations for the minimum content of clinical trial protocols. The Cellular Molecular Pathology Initiative, hosted by the UK National Cancer Research Institute, developed an extension, SPIRIT‐Path, describing how to effectively incorporate pathology support into clinical trial protocols. The current study assessed the inclusion of SPIRIT‐Path items in protocols of active clinical trials. Publicly available clinical trial protocols were identified for assessment against the new guidelines using a single UK hospital as the ‘test site’. One hundred and ninety interventional clinical trials were identified as receiving support from the pathology department. However, only 38 had publicly available full trial protocols (20%) and following application of the inclusion/exclusion criteria, 19 were assessed against the SPIRIT‐Path guidelines. The reviewed clinical trial protocols showed some areas of compliance and highlighted other items that were inadequately described. The latter lacked information about the individuals responsible for the pathology content of the trial protocol, how pathology activities and roles were organised in the trial, where the laboratory work would be carried out, and the accreditation status of the laboratory. Only one trial had information specific to digital pathology, a technology certain to become more prevalent in the future. Adoption of the SPIRIT‐Path checklist will facilitate comprehensive trial protocols that address all the key cellular and molecular pathology aspects of interventional clinical trials. This study highlights once again the lack of public availability of trial protocols. Full trial protocols should be available for scrutiny by the scientific community and the public who participate in the studies, increasing the transparency of clinical trial activity and improving quality.

## Introduction

The SPIRIT (Standard Protocol Items: Recommendations for Interventional Trials) 2013 Statement provides evidence‐based recommendations for the minimum content of clinical trial protocols [1] and has been widely endorsed as an international standard for trial protocols by academia (e.g. *The British Medical Journal*, *The Journal of the American Medical Association*, *The Lancet*) and industry (e.g. GlaxoSmithKline, Janssen, Johnson & Johnson) [2]. The National Cancer Research Institute (NCRI) Pathology Group, formerly the Cellular Molecular Pathology Initiative (CMPath), through a project called SPIRIT‐Path, developed an extension to the original SPIRIT statement describing how to effectively incorporate pathology support into clinical trial protocols. A systematic review of existing guidance for pathology items in clinical trials [3] was used to conduct an international Delphi process from which the SPIRIT‐Path guidelines were derived [4]. The checklist includes 14 items, seven elaborations and seven extensions, to the SPIRIT 2013 Statement that should be addressed in trial protocols with pathology elements. SPIRIT‐Path recommends that clinical trial protocols should document the personnel, processes, and standards for all cellular and molecular pathology components of a trial, including all stages of the specimen pathway, with specific consideration of how to maximise the value of trial data and associated biological samples for further translational studies [4].

This study assessed baseline compliance with the SPIRIT‐Path guidelines [4]; first, to identify pathology items that are consistently overlooked in clinical trial protocols; second, to highlight areas of good practice and provide verbatim examples abstracted from publicly available trial protocols to illustrate how compliance can be achieved.

## Methods

### Context and identifying clinical trials for assessment

The Newcastle upon Tyne Hospitals NHS Foundation Trust is host to one of the UK Experimental Cancer Medicine Centres facilitating early phase clinical trials [5] and one of the five National Institute for Health Research (NIHR) Patient Recruitment Centres in England, established to ‘increase the UK's capacity to deliver late‐phase commercial clinical research’, facilitate ‘commercial clinical research in the National Health Service’, and provide ‘opportunities for patients to benefit from early access to innovation’ [6]. The Newcastle upon Tyne Hospitals is also part of the Newcastle Health Innovation Partners, which was recently accredited as an Academic Health Science Centre. The Department of Cellular Pathology supports clinical trials at Newcastle upon Tyne Hospitals and provided a ‘test site’ to identify pathology‐relevant, publicly available protocols for assessment against the SPIRIT‐Path guidelines. A comprehensive search of the departmental trials was conducted via email communications with the following:

- The Integrated Laboratory Medicine Business Unit for a list of clinical trials held on a Microsoft Access Invoicing Database.
- The laboratory manager of the research arm of Cellular Pathology for a list of active clinical trials supported by the facility.
- Consultant pathologists for information about their involvement in clinical trial work.

### Accessing trial protocols

Trial details were investigated using ScanMedicine [7], which searches 11 clinical trial registries including those recommended by the SPIRIT Group, for example ClinicalTrials.gov database [8], International Standard Randomised Controlled Trials Number (ISRCTN) registry [9], and EU Clinical Trials Register [10]. For trials with acronyms, the full trial name was identified using Newcastle upon Tyne Hospitals Research Database Application (ReDA; Infonetica, Esher, UK) prior to initiating a search. ScanMedicine provides a short synopsis of the trial and a URL link to the corresponding registry where the detailed trial summary and supporting documents are located. Protocols were either located directly on the registry or through the trial website URL link hosted on the registry page. Non‐commercial studies funded by the NIHR were identified using the ‘NIHR Funding and Awards’ search engine [11].

Inclusion criteria:

- Interventional clinical trial.
- Publicly available full trial protocol.
- Active clinical trial (open: recruiting or in follow‐up).

Exclusion criteria:

- Abridged protocols.
- Redacted protocols.
- Journal articles summarising trial design.

### Assessing trial protocols against the SPIRIT 2013 Statement checklist

The full version trial protocols were assessed to determine compliance with the SPIRIT 2013 Statement. The assessment was carried out by the senior author (MR), who co‐led the development of the SPIRIT‐Path guidelines and was familiar with all the items in the SPIRIT 2013 Statement. The items were categorised as included, not included, or not applicable; the latter was defined as an item that was not included but was irrelevant to the study.

### Assessing trial protocols against the SPIRIT‐Path checklist

We developed a search strategy to assess full version trial protocols with the aim of efficiently identifying all relevant pathology items. The search strategy and data collection methods were piloted on six protocols that were not publicly available. Two assessors (PR and MR) independently analysed the protocols against the SPIRIT‐Path checklist. Where interpretation differed, consensus was reached by discussion. The pilot was used to train and calibrate the assessors and to refine the search strategy. The final search strategy included the search criteria from the ‘pathology‐specific terms’ employed in the systematic review of cellular and molecular pathology input into clinical trials [3]. Additional search terms were used to identify individual SPIRIT‐Path extensions and elaborations (Table 1). The items were categorised as fully included, partially included, not included, or not applicable; the latter was defined as an item that was not included but was irrelevant to the study. For the SPIRIT‐Path 9a elaboration, ‘describe where the laboratory work will be carried out and the accreditation status of the laboratory/site’, items that were categorised as ‘fully included’ were then sub‐categorised into ‘explicitly included’ and ‘implicitly included’.

**Table 1:** Search terms used to identify the SPIRIT‐Path elaborations and extensions

SPIRIT‐Path item	Search terms
Search terms adapted from Lim *et al* [3]	patholo*, histolo*, molecular diagnos*, cytolo*, biobank, biological marker, biomarker
5a elaboration	consultant, advisor, scientist
5d elaboration	molecular tumour board, trial management group, data monitoring committee, steering
6a elaboration	background, rationale
9 elaboration	Good Clinical Laboratory Practice, Clinical Laboratory Improvement Amendments, International Organization for Standardization, certificat*, accredit*, laborator*
10 extension	central, double, consensus, review, handling
12 extension	schedule, biopsy
18a (i) extension	Good Clinical Practice, training, trained
18a (ii) extension	schedule, biopsy, specimen, tissue, formalin fixed paraffin embedded
18a (iii) extension	immunohisto*, assay
19 extension	digital, archiv*
20a elaboration^†^	diagnostic drift, interpretation, change
26b elaboration^‡^	consent
31c extension	digital, archiv*
33 elaboration	bioresource, repository, translational, further, additional, future

^†^ Assessment of diagnostic drift was only considered for trials that had a prolonged recruitment phase and/or an extended follow‐up period (>5 years).

^‡^ Enduring consent was only assessed for future translational research. Translational research embedded in the protocol was excluded from assessment of this elaboration.

Data were recorded on an Excel spreadsheet (Microsoft, Redmond, WA, USA). For cancer trials, the area of study was assigned according to the volumes of the ‘WHO Classification of Tumours’ Bluebook series [12].

## Results

**Figure 1:**
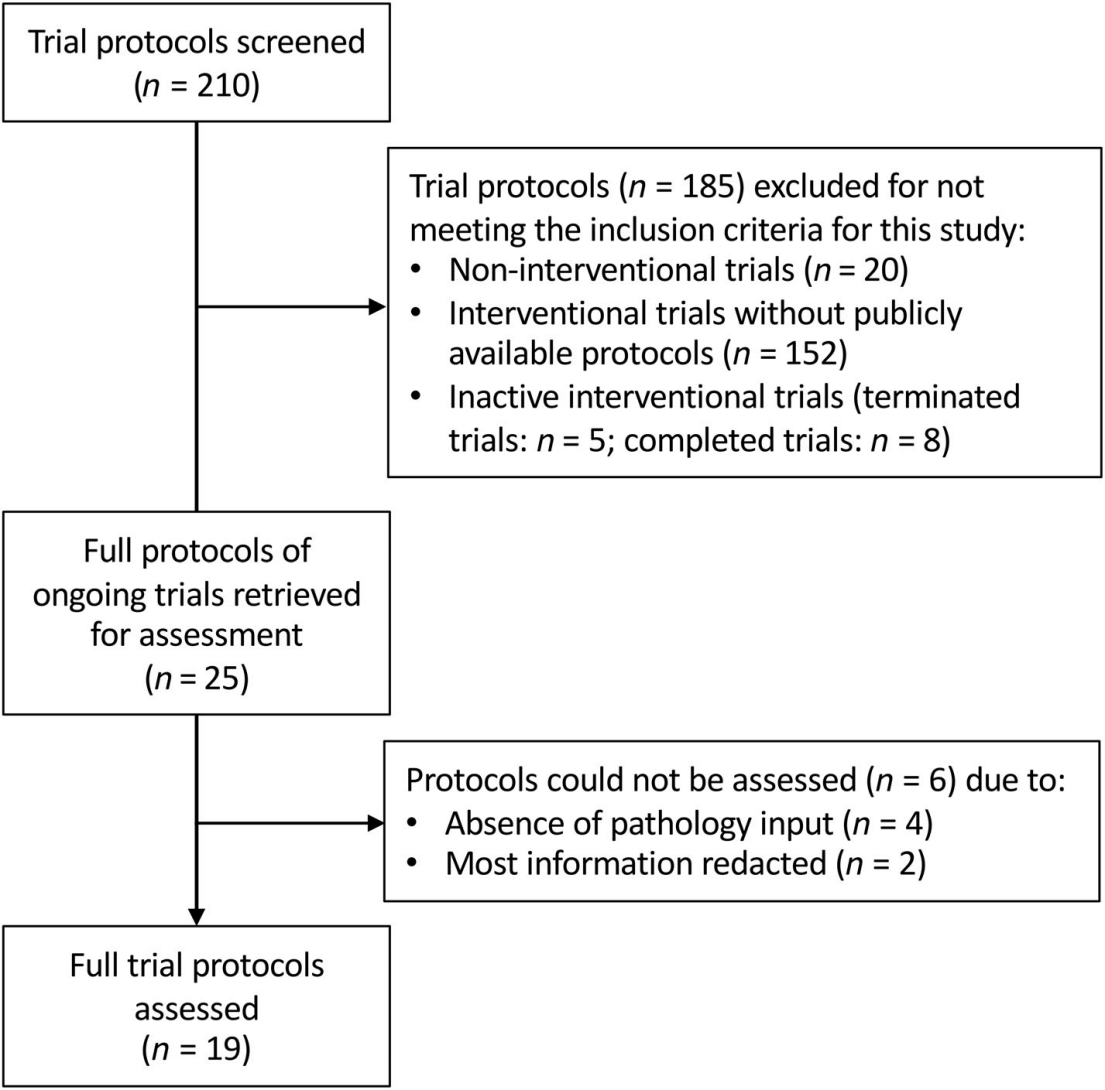
Protocol screening and selection.

Two hundred and ten clinical trials were identified for screening (Figure 1). Following application of the inclusion criteria, there were 25 publicly available clinical trial protocols. However, four had no cellular pathology content and two contained heavily redacted information preventing a full analysis, leaving 19 protocols for assessment against the SPIRIT 2013 Statement and the SPIRIT‐Path guideline (supplementary material, Table S1). Twelve trials were non‐commercial studies and seven were commercial. Protocol publishing dates ranged from 27 July 2015 to 18 April 2021, which were after the SPIRIT 2013 Statement was published [1]. The majority of trials (13/19) were late phase (phase III/IV). Most trials investigated solid tumours (17/19), with breast tumours being the most common (5/17) (Table 2).

**Table 2:** Profile of publicly available trial protocols

Trial	Year	Phase	Disease	Sponsor	References
EuroNet‐PHL‐C2	2015	III	Haematological tumours	Non‐commercial	[13]
LORIS	2016	III	Breast tumours	Non‐commercial	[14]
rEECur	2016	II/III	Soft tissue tumours	Non‐commercial	[15]
ORZORA	2016	IV	Ovarian tumours	Commercial	[16]
ABOUND.2L+	2016	II	Lung tumours	Commercial	[17]
TRIGGER	2017	III	Rectal tumours	Non‐commercial	[18, 19]
SOLO‐1	2018	III	Ovarian tumours	Commercial	[20]
ERA 223	2018	III	Prostate tumours	Commercial	[21]
BOSTON	2018	III	Haematological tumours	Commercial	[22]
FeDeriCa	2018	III	Breast tumours	Commercial	[23]
PHITT	2018	III	Liver tumours	Non‐commercial	[24]
LIFT	2019	IV	Liver transplants	Non‐commercial	[25]
National Lung Matrix Trial	2019	II	Lung tumours	Non‐commercial	[26, 27]
Add‐Aspirin	2019	III	Solid tumours	Non‐commercial	[28]
IMpassion031	2020	III	Breast tumours	Commercial	[29]
OPTIMA	2020	Feasibility	Breast tumours	Non‐commercial	[30]
STAMPEDE	2020	II/III	Prostate tumours	Non‐commercial	[31]
ROAM	2021	III	Central nervous system tumours	Non‐commercial	[32]
SAVER	2021	II	Head and neck tumours	Non‐commercial	[33, 34]

### SPIRIT 2013 Statement checklist

**Figure 2:**
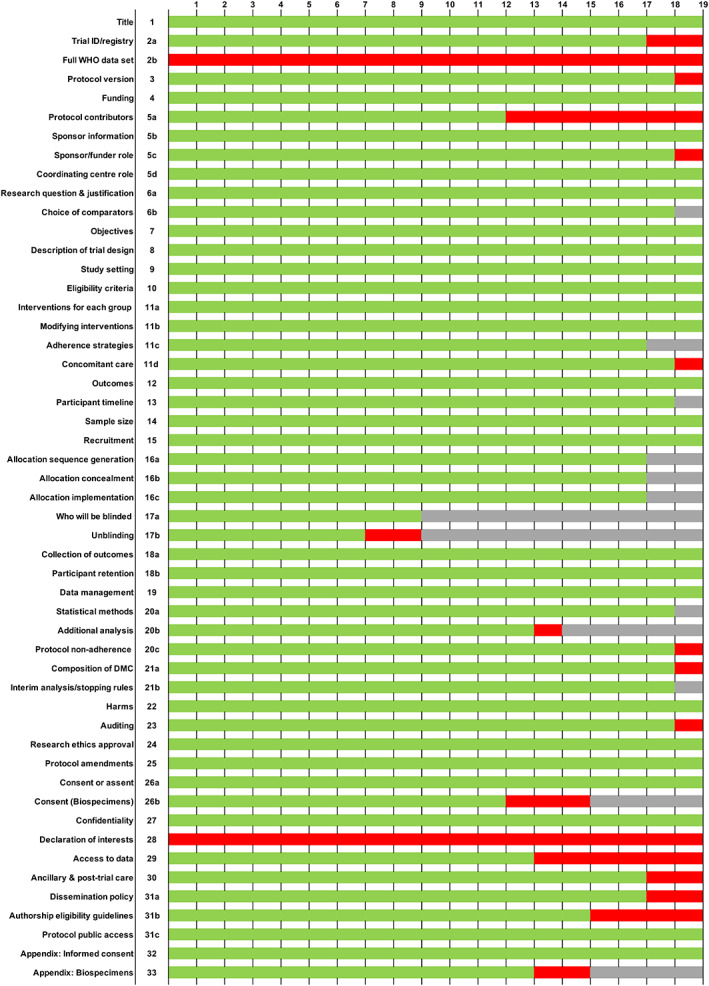
Assessment of publicly available trial protocols against the SPIRIT 2013 Statement. Green, included; red, not included; grey, not applicable.

There was generally good compliance with the SPIRIT 2013 Statement, consistent with protocols written after the publication of guidelines and possibly reflecting publicly accessible documents open to scrutiny by the scientific community and patients (Figure 2). With the exception of the WHO data set (2b) and investigator declaration of interests (28), which were not mentioned in any of the protocols, there were notable omissions around protocol contributors (5a), access to data (29), and authorship eligibility (31b). The lack of disclosure of contributors and authors was mainly a feature of commercial studies (supplementary material, Figure S1).

### SPIRIT‐Path checklist

**Figure 3:**
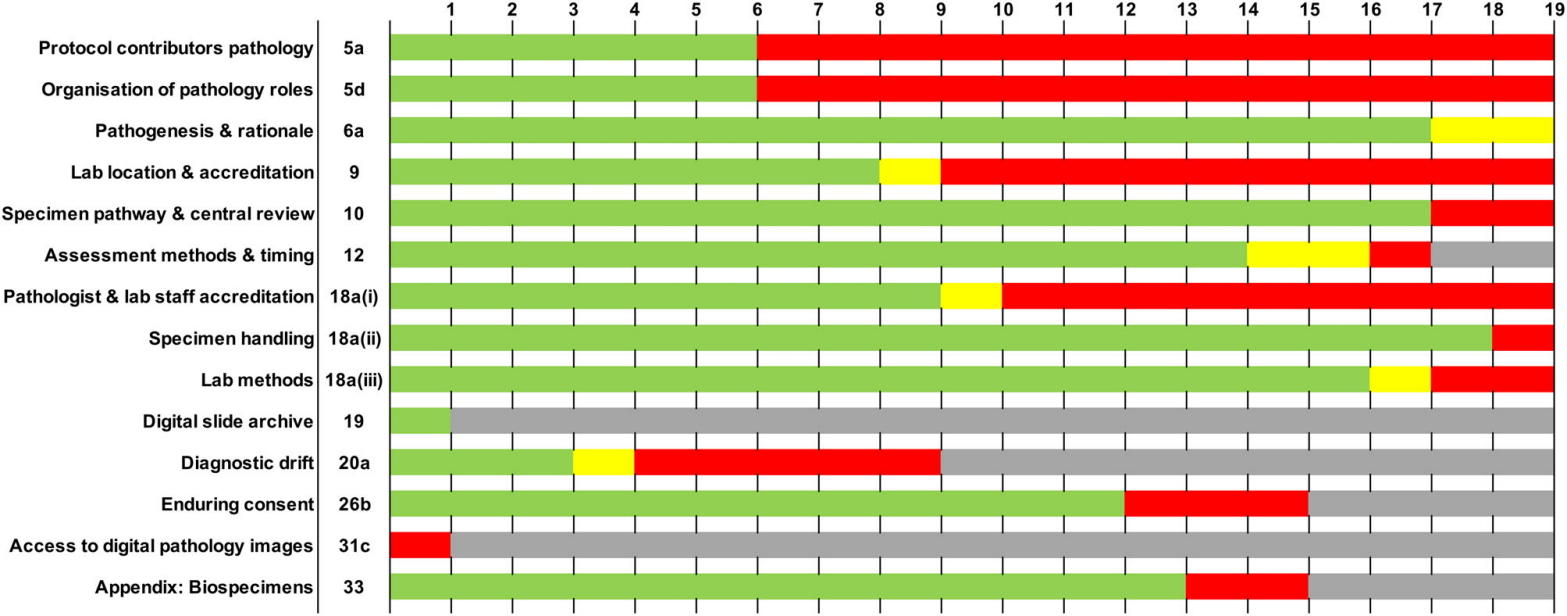
Assessment of publicly available trial protocols against the SPIRIT‐Path items. Green, fully included; yellow, partially included; red, not included; grey, not applicable.

Compliance with the SPIRIT‐Path items is summarised in Figure 3 and supplementary material, Figure S2.

*5a elaboration: Specify the individual(s) responsible for pathology content of the trial protocol and 5d elaboration: Specify how pathology activities and roles are organised in the trial*.

Six trial protocols named the individuals responsible for the pathology content of the protocol and described how pathology activities and roles were organised. The compliant protocols were all non‐commercial trials. By contrast, no commercial studies provided this information. Specifically, there was documentation of a pathologist in the Trial Management Group in all the compliant studies. In addition, one trial included a pathologist on the Trial Steering Committee [14] and another had a designated ‘Pathology Committee’ [24].

*6a elaboration: Describe the pathogenesis of the disease and rationale for any pathology‐specific inclusion criteria or endpoints*.

Seventeen trial protocols described the pathogenesis of the disease being studied and the rationale for pathology‐specific inclusion criteria and/or endpoints. Two trials, one commercial and one non‐commercial, were categorised as ‘partially included’ as they failed to adequately describe pathogenesis, but included pathology relevant inclusion criteria.

*9 elaboration: Describe where the laboratory work will be carried out and the accreditation status of the laboratory/site*.

This elaboration was fully satisfied by eight study protocols: two included explicit accreditation details (‘GCP Lab’; ‘NEQAS performance’) [26, 33] and six trials implied accreditation by documenting the use of hospital laboratories. One trial was categorised as ‘partially included’ as accreditation was implied, but the laboratory location was not disclosed. Ten protocols did not provide sufficient information to satisfy this elaboration. No commercial trials ‘fully included’ this elaboration.

*10 extension: Where trial‐specific pathology reporting is required, document specimen pathway requirements, and any requirement for pathologist ‘double reporting’ or central review*.

The majority of protocols (17 of 19) fully addressed this extension. Twelve documented a requirement for central pathology review (7 of 12 non‐commercial trials and 5 of 7 commercial trials).

*12 extension: Outline the assessment methods and the timing of tissue sampling required for any pathology‐specific outcomes*.

Seventeen trials were assessable for this extension; two trials did not contain pathology‐specific outcomes. Fourteen trials ‘fully included’ the assessment methods and timing for pathology‐specific outcomes. Two trials partially satisfied the extension as they documented the timing of sampling for pathology‐specific outcomes but did not document the assessment methods. One trial outlined a pathology‐specific outcome in the trial design but did not provide sufficient details and was categorised as ‘not included’.

*18a (i) extension: Describe any specific accreditation, training, and performance assessment requirements for trial pathologists and laboratory staff*.

Nine protocols fully satisfied this extension, all stating requirements for investigator Good Clinical Practice (GCP) accreditation. Two documented further investigator requirements, for example, ‘qualified by education, training, and experience to assume responsibility for the proper conduct of the trial at their site and should provide evidence of such qualifications through an up‐to‐date curriculum vitae and/or other relevant documentation’ [31] and ‘all investigators will have the particular medical expertise necessary to conduct the study in accordance to the protocol and all regulatory and ethical requirements’ [33]. All trials documented the requirement for the study to be conducted in line with GCP; however, this was not recorded as compliant because the SPIRIT‐Path extension is specifically related to trial personnel. One trial partially included the extension as there was no mention of investigator GCP accreditation, but there were details on the performance of individuals performing pathology tasks ‘under the supervision of a trained histopathologist’ [26].

*18a (ii) extension: Describe the specimen documentation requirements and full specimen handling pathway*.

Eighteen trials fully included this extension; only one trial did not. Sixteen trial protocols referred to an additional laboratory manual where further information on sampling, sample collection, storage, and shipping procedures was documented.

*18a (iii) extension: Define any methods for specimen assessment by histochemical, immunohistochemical, or molecular techniques*.

Sixteen trials ‘fully included’ this extension. One trial was categorised as ‘partially included’ as it described the assessment methods for pathology‐specific inclusion criteria, but not the pathology‐specific outcome. Two protocols did not document any assessment methods for pathology‐specific activities. All commercial studies ‘fully included’ this extension.

*19 extension: Describe any intended use of a digital pathology slide archive*.

Only one trial was assessable for this extension and ‘fully included’ details of a digital pathology slide archive.

*20a elaboration: Describe any methods to be used for adjusting for diagnostic drift during the trial*.

Diagnostic drift is defined as ‘a gradual change in nomenclature, grading of lesions, or scoring of a biomarker within a single study over time’ [35]. Diagnostic drift was considered assessable for trials with a recruitment phase and/or follow‐up period greater than 5 years. Diagnostic drift was considered to be addressed if pathology assessments were defined by a dated/specific version of assessment criteria or if the trial detailed procedures to mitigate against classifications or assessment methods change. Three of nine trials considered at risk of diagnostic drift ‘fully included’ methods to moderate the effects of this variable; two documented assessment methods restricted to a dated/specific version of assessment and one documented procedures should the tumour classification change in the future. One trial ‘partially included’ this item as it addressed diagnostic drift for recruitment criteria, but not pathology outcome criteria. The remaining five protocols did not report consideration of diagnostic drift.

*26b elaboration: Document enduring consent for future translational studies using tissue or any digital pathology images, if applicable*.

Fifteen trials were assessable for this elaboration. Twelve fully documented enduring consent for future translational studies and three also addressed procedures for the withdrawal of enduring consent.

*31c extension: Describe the mechanism and timing for making digital pathology images available, if applicable*.

Only one trial was assessable for this extension but did not include the mechanism or timing for making digital pathology images available.

*33 elaboration: Specify the regulatory approvals required for clinical trial samples to be used in future work*.

Fifteen trials were assessable for this elaboration, of which 13 ‘fully included’ the required information. Two were categorised as ‘not included’ as they did not document where tissue samples were being stored or approvals for their future use.

## Discussion

This is the first study to assess current clinical trial protocols against the recently published SPIRIT‐Path guidance. Newcastle upon Tyne Hospitals was chosen as a ‘test site’ based on its high recruitment to a broad range of clinical trials; nevertheless, it is conceivable that the process of vetting trials in Newcastle may have led to selection bias in favour of high‐quality trial protocols. To some extent, this is supported by the high levels of compliance of the protocols with the SPIRIT 2013 Statement [1]. Furthermore, the use of a single centre may limit the generalisability of the study findings. Locating and accessing trial protocols was problematic as there are several clinical trial registries and it was not always obvious which registry was likely to host the information. However, during the course of our study we discovered ScanMedicine [7], a search engine containing data uploaded by the National Institute for Health Research Innovation Observatory (NIHRIO). Searches using the full trial name provided reliable signposting for clinical trial protocol information while trial acronyms typically returned numerous results that were sometimes difficult to resolve without further information. Most protocols were deposited on the ClincalTrials.gov registry [8] or the ISRCTN registry [9]. Some trials were registered on both sites and in some of these cases the full protocol was available on one site but not the other, or there was conflicting information regarding protocol versions, recruitment figures, and overall trial status (in set‐up, open, closed, follow‐up). Such discrepancies in trial registry information have been reported previously [36, 37, 38]. Some trials did not have a full protocol deposited on the registry but had links to documents on a study‐specific website. All non‐commercial trials that were funded by the NIHR had protocols that were available on the ‘Funding and Awards’ database [11].

Of the 190 interventional clinical trials screened, only 38 (20%) had publicly available full trial protocols. The lack of patient and public access to clinical trial information has been highlighted previously and raises issues around transparency and scrutiny [39]. The European Medicines Agency (EMA) recently enacted a major change to clinical trial conduct through the Clinical Trials Regulation (Regulation [EU] No 536/2014) which came into application on 31 January 2022. This new legislation mandates increased transparency in trial information and introduces a requirement for ‘information on the authorisation, conduct, and results of each clinical trial carried out in the EU to be publicly available’ [40]. The lack of transparency in clinical trial disclosure is precisely what makes it difficult to determine why they are not publicly available in the first place. The barriers to protocol sharing typically relate to intellectual property rights and when investigators have signed agreements with sponsors or funders that restrict their freedom to disseminate the protocol [39]. At the inception of our study, we contacted sponsors and investigators to request access to the full trial protocols and were surprised to be denied access even to trials that received public funding. Consequently, we decided to focus our study on publicly available protocols and highlight the ‘non‐disclosure’ problem.

Following the application of the inclusion and exclusion criteria, there were 25 publicly available clinical trial protocols available for assessment against the SPIRIT‐Path guidelines. However, four trials had no cellular pathology input and two protocols were so heavily redacted that they could not be assessed. The decision to assess publicly available protocols is likely to have selected for high‐quality trials and consequently our findings cannot necessarily be extrapolated to protocols that are not in the public domain. The consideration of pathology items in the excluded studies could be significantly worse.

Of the 14 SPIRIT‐Path items, 8 (6a, 10, 12, 18a(ii), 18a(iii), 19, 26b, 33) were adequately addressed by the majority of trial protocols. Examples of good practice are provided in supplementary material, Table S2. The following discussion provides a commentary on the items with variable compliance across the studies.

No commercial trials provided a list of investigators or trial personnel. The SPIRIT 2013 Statement recommends that ‘individuals who contribute substantively to protocol development and drafting should have their contributions reported’ and the ‘naming of contributors can also help to identify competing interests and reduce ghost authorship’ [41]. The SPIRIT Statement also states in item 31b that ‘individuals who fulfil authorship criteria should not remain hidden (ghost authorship) and should have final authority over manuscript content’ [41]. ‘Ghost authorship’ or ‘ghost writing’ refers to the failure to designate an individual, in this case an industry employee or an external medical writer, who has made a substantial contribution to the research or writing of an article as an author [42]. The issues of ghost writing, accountability, transparency, and conflicts of interest in clinical trials have been documented previously [43, 44, 45, 46]. Lack of recognition of pathology input into clinical trials is a barrier to pathologists' support [47]. Appropriate acknowledgement of pathologists when reporting clinical trials will encourage more pathologists to support future research endeavours.

The utility of the SPIRIT‐Path extensions is exemplified by areas that showed compliance against the SPIRIT 2013 Statement, but did not satisfy SPIRIT‐Path guidance. For example, while the all the studies were compliant with the SPIRIT 2013 Statement item 9 (‘study set up’), only half described where the laboratory work will be carried out and the accreditation status and no commercial studies provided this information. Furthermore, the majority of protocols that ‘fully included’ this elaboration only implied the accreditation of the laboratory by naming a clinical provider. It would have been more informative to include the accreditation standard (e.g. Clinical Laboratory Improvement Amendments, Good Clinical Laboratory Practice, International Organization for Standardization). Approximately half of the protocols specifically stated investigator GCP accreditation. The decision to judge the remaining trials to be non‐compliant is a moot point as they all documented the requirement for the study to be conducted in line with GCP; however, the SPIRIT‐Path extension is specifically related to trial personnel. There was only one trial assessable for the digital pathology extensions; however, in the future, digital pathology is likely to become more widespread and has the potential to provide greater accuracy, reproducibility, and standardisation of pathology‐based trial entry criteria and endpoints [48].

Disease classification evolves and refinements based on molecular information are increasingly incorporated into clinical practice. Late‐phase clinical trials are particularly prone to changes in classification, grading, and staging information [35]. For example, in the LORIS trial [14], the researchers highlight the potential risk that grading of breast ductal carcinoma *in situ* may evolve from a three‐tier system (low, intermediate, high) to a binary classification (high‐grade versus non‐high grade). To mitigate this, they emphasise the importance of central review of the pathology and indicate that changes to the trial protocol may be required if the evidence base for an alternative grading system emerges. It is worth highlighting that companion diagnostic tests incorporated into trial design are also at risk of changing over time as new tests are adopted and scoring algorithms and decision thresholds are modified. Consideration of diagnostic drift is important, but difficult to predict. A statement recognising the phenomenon with active monitoring over the course of the study is sufficient to ‘future proof’ studies that are subject to long recruitment phases and prolonged follow‐up periods.

In conclusion, the publicly available clinical trial protocols published prior to the SPIRIT‐Path guidelines show areas of good compliance and items that are inadequately described. The SPIRIT‐Path guidelines, first and foremost, encourage early engagement of clinical trial protocol writers with pathologists and laboratory scientists. The extensions and elaborations provide both trialists and the pathology community with sufficient guidance to be able to incorporate precise information into protocols thereby facilitating effective trial implementation. The guidance emphasises the importance of planning molecular pathology eligibility criteria, pathology‐specific end points, and the role of the laboratory in translational research. We encourage industry, academics, sponsors, and funders to adopt SPIRIT‐Path to produce comprehensive interventional clinical trial protocols. Furthermore, SPIRIT‐Path, along with SPIRIT‐PRO and SPIRIT‐AI [49, 50], provide models to leverage contributions and expertise from under‐represented clinical trial stakeholders. The study highlights once again the lack of publicly available trial protocols [39]. We believe full trial protocols should be available for scrutiny by the scientific community and the public who participate in the studies and contribute funding through taxation and charitable donations.

## Author contributions statement

MR and TJK conceived the study. All the authors contributed to the development of the protocol. MR and PR extracted, analysed and synthesised the data. MR and PR wrote the first draft of the manuscript, and all authors critically reviewed and approved the final version of the manuscript.

## Supporting information


**Figure S1.** Assessment of publicly available trial protocols against the SPIRIT 2013 Statement with sub‐grouping by commercial and non‐commercial trials
**Figure S2.** Assessment of publicly available trial protocols against the SPIRIT‐Path items with sub‐grouping by commercial and non‐commercial trials
**Table S1.** Details of the clinical trial protocols assessed
**Table S2.** Examples of where SPIRIT‐Path items were fully addressed
