# Supplementary material for: Assessment of clinical trial protocols for pathology content using the SPIRIT‐Path guidelines highlights areas for improvement
Source: J Pathol Clin Res. 2022 May 31;8(5):411–21. doi: 10.1002/cjp2.274 (PMC9353656; doi:10.1002/cjp2.274)
Supplement: Supplementary file 1 — Figure S1. Assessment of publicly available trial protocols against the SPIRIT 2013 Statement with sub‐grouping by commercial and non‐commercial trials Figure S2. Assessment of publicly available trial protocols against the SPIRIT‐Path items with sub‐grouping by commercial and non‐commercial trials Table S1. Details of the clinical trial protocols assessed Table S2. Examples of where SPIRIT‐Path items were fully addressed [file CJP2-8-411-s001.pdf]

Assessment of clinical trial protocols for pathology content using the SPIRIT-Path guidelines highlights areas for improvement

P Robinson *et al*. *J Pathol Clin Res* DOI: 10.1002/cjp2.274

Supplementary Material

Supplementary Figures S1 and S2

Supplementary Tables S1 and S2

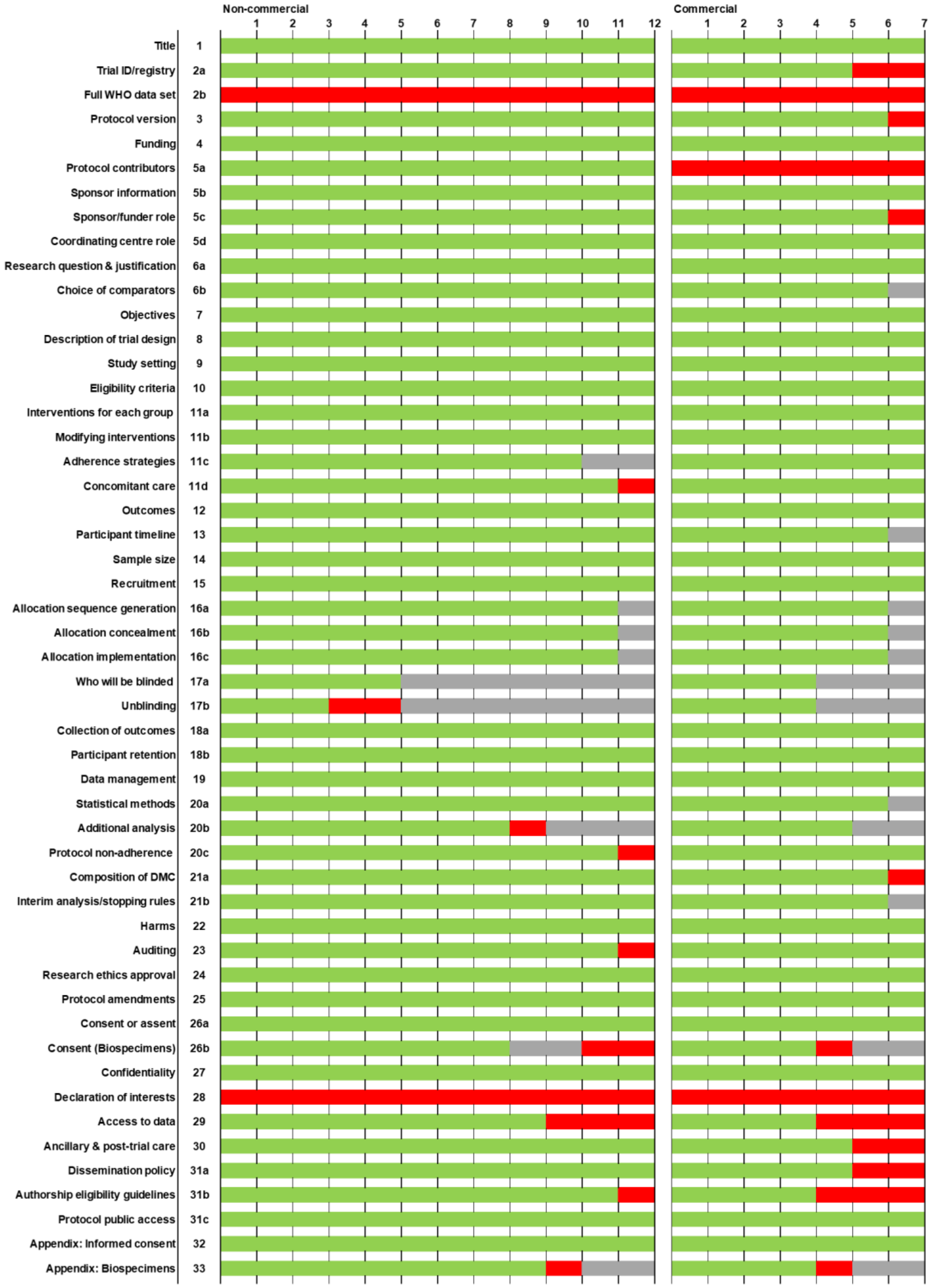

Figure S1. Assessment of publicly available trial protocols against the SPIRIT 2013 Statement with sub-grouping by commercial and non-commercial trials. Key: green = included, red = not included, grey = not applicable.

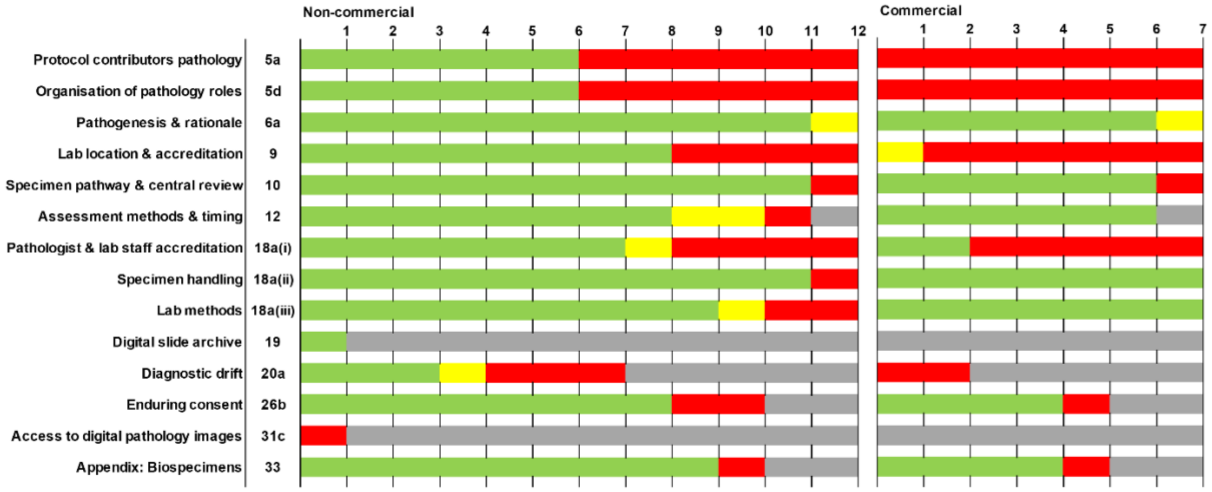

Figure S2. Assessment of publicly available trial protocols against the SPIRIT-Path items with sub-grouping by commercial and non-commercial trials. Key: green = fully included, yellow = partially included, red = not included, grey = not applicable.

Table S1. Details of the clinical trial protocols assessed.

Trial name (acronym)	Authors	Version [date]	Sponsor	Trial number	Available from [accessed]
European Network of Paediatric Hodgkin Lymphoma Second International Inter-Group Study for Classical Hodgkin Lymphoma in Children and Adolescents (EuroNet-PHL-C2)	Körholz D, Hamish Wallace W, Landman-Parker J, *et al*.	Final [27/07/2015]	University of Giessen	NCT02684708	https://www.skion.nl/workspace/uploads/EuroNet-PHL-C2_trial_protocol_final2-0_2015-07-27.pdf [07/09/2021]
A Phase III Trial of Surgery versus Active Monitoring for Low Risk Ductal Carcinoma in Situ (DCIS) (LORIS)	Francis A, Rea D, Fallowfield L, *et al*.	4.0 [15/03/2016]	University of Birmingham	ISRCTN27544579	https://www.birmingham.ac.uk/research/crctu/trials/loris/index.aspx [07/09/2021]
rEECur: an international randomised controlled trial of chemotherapy for the treatment of recurrent and primary refractory Ewing sarcoma	McCabe M, Wheatley K, Whelan J, *et al*.	5.0 [03/06/2016]	University of Birmingham	ISRCTN36453794	https://www.birmingham.ac.uk/research/crctu/trials/reecur/index.aspx [07/09/2021]

(rEECur)					
An Open Label, Single Arm, Multicentre Study to Assess the Clinical Effectiveness and Safety of Lynparza (Olaparib) Capsules Maintenance Monotherapy in Platinum Sensitive Relapsed Somatic or Germline BRCA Mutated Ovarian Cancer Patients Who Are in Complete or Partial Response Following Platinum Based Chemotherapy (ORZORA)	AstraZeneca	3 [22/07/2016]	AstraZeneca	NCT02476968	https://www.clinicaltrials.gov/ct2/show/NCT02476968 [07/09/2021]
A Phase 2, Open-Label, Multi-Centre Study to Assess Safety and Efficacy of Second/Third-Line Treatment With NAB®-Paclitaxel	Celgene Corporation	5.0 [09/12/2016]	Celgene Corporation	NCT02250326	https://clinicaltrials.gov/ct2/show/study/NCT02250326 [07/09/2021]

(ABI-007) In Combination With Epigenetic Modifying Therapy Of CC-486, Or Immunotherapy of Durvalumab (MEDI4736), Or As Monotherapy In Subjects With Advanced Non-Small Cell Lung Cancer (NSCLC) (ABOUND.2L+)					
Magnetic Resonance Tumour Regression Grade (mrTRG) as a Novel Imaging Biomarker to Stratify Management of Good and Poor Responders to Chemoradiotherapy: A Rectal Cancer Multicentre Randomised Control Trial (TRIGGER)	Brown G, Rao S, Khakoo S, *et al*.	5.0 [07/03/2017]	Royal Marsden NHS Foundation Trust	NCT02704520	https://www.ncbi.nlm.nih.gov/pmc/articles/PMC5576102/ [07/09/2021]

A Phase III, Randomised, Double Blind, Placebo Controlled, Multicentre Study of Olaparib Maintenance Monotherapy in Patients With BRCA Mutated Advanced (FIGO Stage III-IV) Ovarian Cancer Following First Line Platinum Based (SOLO-1)	AstraZeneca	Not assessable [21/02/2018]	AstraZeneca	NCT01844986	https://clinicaltrials.gov/ct2/show/NCT01844986 [07/09/2021]
A Phase III Randomised, Double-blind, Placebo-controlled Trial of Radium-223 Dichloride in Combination With Abiraterone Acetate and Prednisone/Prednisolone in the Treatment of Asymptomatic or Mildly	Bayer HealthCare Pharmaceuticals Inc	5.0 [03/04/2018]	Bayer HealthCare Pharmaceutical Inc	NCT02043678	https://www.clinicaltrials.gov/ct2/show/NCT02043678 [07/09/2021]

Symptomatic Chemotherapy-naïve Subjects With Bone Predominant Metastatic Castration-resistant Prostate Cancer (CRPC) (ERA 223)					
A Phase 3 Randomised, Controlled, Open-label Study of Selinexor, Bortezomib, and Dexamethasone (SVd) Versus Bortezomib and Dexamethasone (Vd) in Patients With Relapsed or Refractory Multiple Myeloma (RRMM) (BOSTON)	Karyopharm Therapeutics Inc	4.0 [17/08/2018]	Karyopharm Therapeutics Inc	NCT03110562	https://clinicaltrials.gov/ct2/show/NCT03110562 [07/09/2021]
A Phase III, Randomised, Multicentre, Open-Label, Two-Arm Study to Evaluate the Pharmacokinetics,	F. Hoffmann-La Roche Ltd	2 [12/10/2018]	F. Hoffmann-La Roche Ltd	NCT03493854	https://clinicaltrials.gov/ct2/show/NCT03493854 [07/09/2021]

Efficacy, and Safety of Subcutaneous Administration of the Fixed-Dose Combination of Pertuzumab and Trastuzumab in Combination with Chemotherapy in Patients with HER2-Positive Early Breast Cancer (FeDeriCa)					
Paediatric Hepatic International Tumour Trial (PHITT)	Morland B, Wheatley K, Dandapani M, *et al*.	3.0 [17/10/2018]	University of Birmingham	NCT03017326	https://www.birmingham.ac.uk/research/crctu/trials/phitt/index.aspx [07/09/2021]
Prospective Randomised Marker-based Trial to Assess the Clinical Utility and Safety of Biomarker-guided Immunosuppression Withdrawal in Liver Transplantation (LIFT)	Sanchez-Fueyo A, Leithead J, Tachtatzis P, *et al*.	11 [27/09/2019]	King’s College Hospital NHS Foundation Trust, King’s College London	NCT02498977	https://fundingawards.nihr.ac.uk/award/13/94/55 [07/09/2021]

National Lung Matrix Trial: Multi-drug, Genetic Marker-directed, Non-comparative, Multi-centre, Multi-arm Phase II Trial in Non-small Cell Lung Cancer (National Lung Matrix Trial)	Middleton G, Billingham L, Savage J, *et al*.	8.0 [18/10/2019]	University of Birmingham	NCT02664935	https://www.nature.com/articles/s41586-020-2481-8 [07/09/2021]
A Phase III, Double-blind, Placebo-controlled, Randomised Trial Assessing the Effects of Aspirin on Disease Recurrence and Survival After Primary Therapy in Common Non Metastatic Solid Tumours (Add-Aspirin)	Langley R, Dibble T, Robbins A, *et al*.	7.0 [21/10/2019]	University College London, Tata Memorial Centre	ISRCTN74358648	https://fundingawards.nihr.ac.uk/award/12/01/38 [07/09/2021]
A Phase III Randomised Study to Investigate the Efficacy and Safety of Atezolizumab	F. Hoffmann-La Roche Ltd	7 [11/02/2020]	F. Hoffmann-La Roche Ltd	NCT03197935	https://clinicaltrials.gov/ct2/show/NCT03197935 [07/09/2021]

(Anti-PD-L1 Antibody) in Combination With Neoadjuvant Anthracycline/Nab-Paclitaxel-Based Chemotherapy Compared With Placebo and Chemotherapy in Patients With Primary Invasive Triple-Negative Breast Cancer (IMpassion031)					
Optimal personalised treatment of early breast cancer using multiparameter analysis (OPTIMA)	Stein R, Makris A, Hughes-Davies L, *et al*.	7.0 [11/09/2020]	University College London	ISRCTN42400492	https://fundingawards.nihr.ac.uk/award/10/34/501 [07/09/2021]
Systemic Therapy in Advancing or Metastatic Prostate Cancer: Evaluation of Drug Efficacy: A multi-arm multi-stage randomised	Parker C, Attard G, Clarke N, *et al*.	21.0 [20/10/2020]	University College London	NCT00268476	https://www.ctu.mrc.ac.uk/studies/all-studies/s/stampede/ [07/09/2021]

controlled trial (STAMPEDE)					
Radiation versus Observation following surgical resection of Atypical Meningioma: a randomised controlled trial (ROAM)	Jenkinson M, Javadpour M, Haylock B, *et al*.	5.0 [18/03/2021]	The Walton Centre NHS Foundation Trust	ISRCTN71502099	https://fundingawards.nihr.ac.uk/award/12/173/14 [07/09/2021]
Sodium valproate for epigenetic reprogramming in the management of high risk oral epithelial dysplasia (SAVER)	Shaw D, McCarthy C, Sacco J, *et al*.	9.0 [19/04/2021]	University of Liverpool	ISRCTN12448611	https://fundingawards.nihr.ac.uk/award/14/209/13 [07/09/2021]

Table S2. Examples of where SPIRIT-Path items were fully addressed.

SPIRIT-Path item	Example	Trial acronym[1]
5a. Specify the individual(s) responsible for pathology content of the trial protocol.	'Biology & Pathology Coordinators<br>Dr Carolina Armengol, Dr Rita Alaggio, Prof Gareth Veal, Dr Torsten Pietsch, Dr Stefano Cairo, Prof Roland Kappler, Dr Julia von Frowein.' [P7]	PHITT
5d. Specify how pathology activities and roles are organised in the trial.	'23.3 Trial Management Group<br>The TMG is composed of the Chief Investigator, co-investigators, representatives from each NCC, biology and pathology committee and the trial team at the CRCTU. The TMG is responsible for the day-to-day running and management of the trial and will meet by teleconference or in person at least every 3 months.' [P98]	PHITT
6a. Describe the pathogenesis of the disease and rationale for any pathology-specific inclusion criteria or endpoints.	*Pathogenesis*<br>'Triple-negative breast cancer (TNBC) is defined by the absence of immunostaining for oestrogen receptor (ER), progesterone receptor (PgR), and human epidermal growth factor receptor 2 (HER2). Overall, approximately 15%–20% of all breast cancers are classified as TNBC. Large-scale comprehensive genomic analyses have characterised the heterogeneous nature of TNBCs and their diverse gene expression patterns and underlying genomic changes, but these insights have not yet provided clear guidance for the identification of clinically effective targeted therapies currently under laboratory and clinical investigation. Unfortunately, TNBCs are more likely to have aggressive features, such as a high proliferative rate, and exhibit an invasive phenotype. Patients with TNBCs exhibit a poor clinical outcome, generally with rapid progression and a shorter time to local and distant relapse (Dent et al. 2007).' [P26].<br><br>*Pathology-specific inclusion criteria*<br>'Inclusion Criteria<br>Histologically documented TNBC (negative HER2, ER, and PgR status); HER2 negativity will be defined by central laboratory assessment using ISH or IHC	IMpassion031

	assays per ASCO/CAP criteria and ER/PgR negativity will be defined by central laboratory assessment using IHC per ASCO/CAP criteria. Central laboratory assessment will occur prior to randomisation.<br>Patients with multifocal tumours (more than one tumour confined to the same quadrant as the primary tumour) are eligible provided all discrete lesions are sampled and centrally confirmed as TNBC.<br>• Confirmed tumour PD-L1 evaluation as documented through central testing of a representative tumour tissue specimen<br>In Stage 2, if the iDMC recommendation is to expand to a PD-L1–positive population, only patients with confirmed tumour PD-L1 positive (IC1/2/3) will be considered eligible.<br>• Primary breast tumour size of > 2 cm by at least one radiographic or clinical measurement<br>• Stage at presentation: cT2–cT4, cN0–cN3, cM0'. [P17].<br><br>*Pathology-specific inclusion criteria rationale*<br>'3.3.2 Rationale for Patient Population and Analysis Groups<br>Patients with cT2–cT4d TNBC were selected for this study because increased primary tumour size has been identified as poor prognostic variables and has been associated with decreased disease-free survival (DFS) and increased likelihood of early metastatic disease in patients with TNBC (Pistelli et al. 2013; Rosa Mendoza et al. 2013). Not-yet-published internal analyses from the California Cancer Registry found survival rates in patients with Stage II or III disease to be significantly lower than those of patients diagnosed with Stage I disease (3-year OS rate of 94.2% [95% CI: 93.3, 95.0] for Stage I; 86.2% [95%CI: 85.1, 87.2] for Stage II; and 58.8% [95%CI: 56.3, 61.2] for Stage III; data on file). By selecting a patient population that has an increased rate of disease recurrence (larger tumour size) and poorer clinical outcomes and by further enriching this with patients with node-positive disease, the study will enrol patients with TNBC who have the highest unmet medical need.' [P40].<br><br>*Pathology endpoint*	

	‘The primary efficacy endpoint (pathologic complete response [pCR]; ypT0/is ypN0) will be established via local review following completion of neoadjuvant therapy and surgery. Pathologists who review study specimens must utilise the evaluations and assessments outlined in the Pathology Manual’ [P36].<br><br>*Pathology endpoint rationale*<br>‘Rationale for Pathologic Complete Response as Primary Endpoint<br>pCR was selected as the primary efficacy endpoint; it is a validated, meaningful measure of response to therapy, and on the basis of data from several analyses and clinical trials and meta-analyses, there is an association between the pCR status of a patient and long-term outcomes (Cortazar et al. 2014; Liedtke et al. 2008; von Minckwitz et al. 2012). This association was especially strong in patients with TNBC (EFS hazard ratio [HR] 0.24 [95% CI: 0.18, 0.33]; OS HR 0.16 [95% CI: 0.11, 0.25]) (Cortazar et al. 2014).’ [P43].	
9. Describe where the laboratory work will be carried out and the accreditation status of the laboratory/site.	**‘**Technology Hub<br>Laboratory that performs the NGS testing for both SMP2 and the National Lung Matrix Trial. All SMP2 clinical hubs are paired with one of 3 laboratories – Birmingham, Cardiff and ICR.’ [P353].<br><br>‘Locally obtained genetic results from approved laboratories, may be used to confer molecular eligibility for certain selected genes that fail testing on the SMP2 NGS panel. Approval will be based, in part, on NEQAS performance. Please refer to the current version of the document entitled ‘Use of Local Test Result for SMP2/National Lung Matrix Trial’ for further information on approved laboratories and accepted genes.’ [P25]	National Lung Matrix Trial
10. Where trial-specific pathology reporting is required, document specimen pathway requirements and any requirement for pathologist	’10.4 CENTRAL LABORATORY PROCEDURES<br>The Central Laboratory will in the first instance assess the block(s) for invasive tumour content irrespective of randomisation. If any tissue block is deemed as insufficient or unsuitable a further tissue block will be requested from the recruiting site via the OPTIMA Trial Office.<br>For patients randomised to test-directed treatment, the Central Laboratory will either perform or despatch tissue to a second laboratory for Prosigna testing. The	OPTIMA

'double reporting' or central review.	laboratory will inform the Trial Office of the result of the Prosigna test(s) if performed or if suitable tumour cannot be obtained from the recruiting site. In the ordinary course of events, the laboratory will make 2 attempts to obtain suitable tissue and/or perform a Prosigna test.<br>A small (estimated as approximately 4%, from OPTIMA *prelim*) proportion of patients may require confirmation of tumour ER and HER2 status because of the Prosigna test result, most commonly because the tumour has a non-luminal phenotype. The Central Laboratory will perform receptor re- testing in such cases. ’ [P40]<br><br>’10.3 TUMOUR BLOCK SELECTION AND DOCUMENTATION<br>The collection and subsequent testing of an archival tumour block is integral to patient care in OPTIMA. A suitable tumour block should be sent without delay to the Central Laboratory following patient randomisation, target within 3 working days.<br>Tumour block selection should be performed as follows:<br>• Patients with a unifocal tumour: a representative tumour block should be selected.<br>• Patients who have received pre-operative endocrine treatment: a pre-treatment core biopsy should be selected.<br>A tumour block from a surgical excision or other on-treatment biopsy is not acceptable: treated tumours are likely to have a lower Prosigna Score than untreated tumours, which could change the treatment allocation.<br>• Patients with multiple ipsilateral tumours: blocks from more than one lesion should be submitted to the laboratory when the lesions are considered to be clinically significant by the referring site and they are interpreted as synchronous primary cancers (based either on the site of the lesions, i.e. in different quadrants, or if they are of differing morphology, i.e. histological type or grade). It is anticipated that laboratories will, as per standard good practice, assess ER and HER2 on the different lesions.’ [P39]<br><br>‘Figure 2. Tissue handling and treatment allocation flow diagram’ [P36]	

12. Outline the assessment methods and the timing of tissue sampling required for any pathology-specific outcomes.	*Timing of tissue sampling*<br>'10.8 Liver Biopsies<br>Protocol liver biopsies will be performed:<br>1. At screening (to determine eligibility for study participation and to measure the transcriptional biomarker of tolerance);<br>2. 12 and 24 months after complete IS discontinuation for all recipients in Arms A and B+ who successfully withdraw IS;<br>Patients who completely discontinue IS but require IS reinstitution on the basis of the 12-month post-withdrawal liver biopsy findings will still undergo a follow-up biopsy 24 months after IS withdrawal. In addition, "for-cause" biopsies will be performed to evaluate allograft dysfunction.' [P32].<br><br>*Assessment methods*<br>'24.4 Appendix 4: Processing of formalin-fixed and paraffin embedded liver biopsy samples<br>The following protocol has been designed with the purpose of facilitating the diagnostic assessment at the local centre and the following central pathology review at King's (College Hospital Liver Histopathology laboratory)…<br><br>Fixation and embedding:<br>Formalin fixation and paraffin embedding should be carried out according to the standard local procedure. There is no particular preference for the type of formalin to be used (10 % formal-saline or 10% neutral buffered formalin).<br><br>Sectioning of liver biopsy samples for diagnosis and trial:<br>Trim away excess paraffin wax from around tissue to give a *mesa* shape to the block – this helps with ribboning for cutting serial sections<br>Cut 20 serial sections at 4 microns, and pick up 2 sections per slide<br>Stain as follows:<br>Slide 1: H&E<br>Slide 2: Perls<br>Slide 3: Unstained	LIFT

	Slide 4: Reticulin stain - untoned<br>Slide 5: Masson trichrome<br>Slide 6: Orcein<br>Slide 7: Unstained<br>Slide 8: Diastase Periodic acid Schiff<br>Slide 9: Unstained<br>Slide 10: H&E ... Unstained sections may be used for additional staining or IHC if required.' [P64] – assessment of screening<br><br>'Liver Tissue Tolerance Biomarker<br>'A combination of 5 out of the 10 genes measured at baseline (i.e. before IS was discontinued) was extremely accurate at discriminating those liver recipients who could successfully withdraw IS from those who could not (17). This predictive signature contained the following 5 genes: *SOCS1, TFRC, PEBP1, MIF, CDHR2*, and predicted the outcome of IS withdrawal with AUC=0.85, SN=89%, SP=86%, PPV=80%, and NPV=92%. The signature was different from those reported from PBMCs or whole blood and was highly reproducible across the 3 participating clinical sites. Thus, the test was originally identified in the 48 liver transplant recipients enrolled in Barcelona, and validated in an independent cohort of 21 recipients from Brussels and Rome (17).<br>In order to confirm the reproducibility of the real-time PCR gene expression results originally performed in Hospital Clinic Barcelona in 2011 (Bohne et al. *J Clin Invest* 2012), we conducted a number of additional transcriptional experiments employing the same Applied Biosystems 7900HT real-time PCR platform selected to conduct the current clinical trial. The experiments included a number of commercial and non-commercial RNA calibrators, as well as several different housekeeping genes. Reproducibility was optimised by employing a commercial RNA calibrator (liver RNA, Clontech) and both GAPDH and HPRT1 as housekeeping genes. These experiments were used to re-calibrate the predictive algorithm employing the same exact set-up that will be used in the clinical trial.' [P14].	

18a(i). Describe any specific accreditation, training and performance assessment requirements for trial pathologists and laboratory staff.	‘3.1.1 Principle Investigator’s Qualifications & Agreements<br>1. The investigator(s) should be qualified by education, training, and experience to assume responsibility for the proper conduct of the trial at their site and should provide evidence of such qualifications through an up-to-date curriculum vitae and/or other relevant documentation requested by the Sponsor, the REC, the IRB, and/or the regulatory authorities…<br>4. The investigator should be aware of, and should comply with, the principles of GCP and the applicable regulatory requirements. A record of GCP training should be accessible for all investigators.’ [P34]	STAMPEDE
18a(ii). Describe the specimen documentation requirements and full specimen handling pathway.	‘6.12. Tumour Tissue Sample Collection and Peripheral Blood Collection (Exploratory Assessments)…<br>If a subject’s previously collected tumour tissue (also known as archival tumour) is available, these samples will be collected for biomarker testing as detailed below at Cycle 1 Day 1. If such samples do not exist, have been depleted, or do not contain sufficient tumour material to be analysed, this will not preclude participation in the study and a new biopsy or other procedure to collect tumour tissue will not be required. Table 5 delineates the specific requirements for commonly encountered archival tumour types. Table 5: Requirements for Collection Based on Archival Tumour Sample Type…Details regarding the collection, storage, and shipment of the samples are given in the Laboratory Manual. [P46-47]	ABOUND.2L+
18a(iii). Define any methods for specimen assessment by histochemical, immunohistochemical or molecular techniques.	‘4.5.3 HER2 Screening for Eligibility and Central Assessment of Hormone Receptor Status<br>Patients should be initially screened for HER2 status by the local laboratory and should have an HER2 score of 3+ by IHC or HER2 (c-erbB2) gene amplification by ISH (i.e., fluorescence in situ hybridisation [FISH], silver in situ hybridisation [SISH], or chromogenic in situ hybridisation [CISH]) to qualify for central laboratory screening (see Figure 3). or central confirmation, HER2 positivity is defined as IHC 3 + in $>$ 10% of immunoreactive cells or c-erbB2 gene amplification by ISH (ratio of c-erbB2 gene signals to centromere 17 signals $\geq$ 2.0).	FeDeriCa

	Central laboratory confirmation of a positive HER2 status is required prior to enrolment in the study. The outcome of this assessment will be communicated to the investigator.<br>In addition, central assessment of hormone receptor status (ER and PgR) will be conducted according to ASCO/College of American Pathologists guidelines (Hammond et al. 2010).' [P80-81]<br><br>'Following completion of neoadjuvant therapy and surgery, pCR (ypT0/is, ypN0) will be established via local review. Pathologists who review study specimens must utilise the evaluations and assessments outlined in the Pathology Manual. Further details regarding pathology evaluation and assessment are outlined in the Pathology Manual' [P76].<br><br>'Appendix 10 Pathology Manual [P182-P196].	
19. Describe any intended use of a digital pathology slide archive.	'A full set of H and E stained glass slides and specimen photographs will be centrally reviewed and digitised at the Department of Pathology & Tumour Biology, Leeds Institute of Cancer and Pathology, University of Leeds, UK, the digitised pathology sections will then be archived on the BRC imaging and genomic profiling corelab library server. Central review at Leeds will ensure quality control – quality of surgery and analysis of potential response biomarkers such as ki67, tumour cell density.' [P80]	TRIGGER
20a. Describe any methods to be used for adjusting for diagnostic drift during the trial.	'1.1.3 Classification of Ductal Carcinoma in situ and Consistency of Categorisation It is well recognised that grading of DCIS by pathologists is inconsistent, as shown in the NHSBSP pathology EQA scheme (17). Therefore, all locally diagnosed low and intermediate grade biopsies will be centrally reviewed… If future pathological classification changes to describe high grade and non-high grade DCIS based on robust reproducible criteria rather than the current subjective low/intermediate/high, then the role of active monitoring in the entire non-high grade group may be considered.' [P3]	LORIS
26b. Document enduring consent for future translational studies using	'5.7.3. Collection of tumour sample for exploratory analysis at screening and disease progression (optional)	ORZORA

tissue or any digital pathology images, if applicable.	All patients enrolled will be asked at screening to provide consent for optional most recent archival tumour tissue sample for exploratory analysis.' [P62].<br><br>'5.7.11 Withdrawal of Informed Consent for donated biological samples<br>If a patient withdraws consent to the use of donated biological samples, the samples will be disposed of/destroyed, and the action documented. If the samples are already analysed AstraZeneca is not obliged to destroy the results of this research…..The PI:<br>• Ensures patients' withdrawal of informed consent to the use of donated samples is notified immediately to AstraZeneca or designated Contract Research Organisation (CRO).<br>• Ensures that biological samples from that patient, if stored at the study site, are immediately identified, disposed of /destroyed, and the action documented.<br>• Ensures the laboratory(ies) holding the samples is/are informed about the withdrawn consent immediately and that samples are disposed of/destroyed, the action documented and the signed document returned to the study site.<br>• Ensures that the patient and AstraZeneca are informed about the sample disposal.<br>AstraZeneca ensures the central laboratory(ies) holding the samples is/are informed about the withdrawn consent immediately and that samples are disposed of/destroyed and the action documented and returned to the study site.' [P65-66]	
31c. Describe the mechanism and timing for making digital pathology images available, if applicable.	No examples available.	
33. Specify the regulatory approvals required for clinical trial samples to be used in future work.	'9.2 SAMPLE COLLECTION FOR FUTURE TRANSLATIONAL RESEARCH<br>The Add-Aspirin bio-bank will provide a unique collection of samples with prospective and systematically collected randomised data on aspirin use, cancer outcomes and other potential healthcare benefits related to aspirin. It is intended	Add-Aspirin

	that the bio-bank will be used for a number of translational studies that will be developed and introduced during the trial, subject to securing the appropriate funding. Studies will be reviewed by the TSC and the ethics committee prior to initiation. The tissue collection will also be made accessible to other research groups via application to an Access Committee.<br>The bio-bank is jointly hosted by three Institutions: Tayside Tissue Bank and the Wales Cancer Bank in the UK, and the Advanced Centre for Treatment, Research and Education in Cancer (ACTREC) in India.' [P86]<br><br>6C) Storage and analysis of samples<br>Any tumour samples not used in POLEM translational studies, will be sent to the Add-Aspirin biobanks for use in future Add-Aspirin translational research.' [P162]	

[1]The details of the trials and URL links to the protocols are provided in Table S1.
